# Tales of rationality: the rational emotive behavioral monomyth as a metaphorical alignment of rational emotive behavior therapy and the Hero’s Journey narrative structure

**DOI:** 10.3389/fpsyg.2025.1572636

**Published:** 2025-06-02

**Authors:** Martin J. Turner

**Affiliations:** Department of Psychology, Manchester Metropolitan University, Manchester, United Kingdom

**Keywords:** CBT, REBT, story, narrative, metaphor

## Abstract

In the present paper, a metaphor is proposed for the depiction of rational emotive behavior therapy (REBT) with a view to aiding practitioner and client comprehension of the fundamental processes of REBT. Specifically, it is argued that the processes, and broad framework, of REBT is akin to an archetypal narrative story structure known as the Hero’s Journey, or monomyth. In the Hero’s Journey, an ordered narrative framework is followed that presents the heroic story of a protagonist, a device that has been employed across art, literature, film, and marketing. In the present paper, this Hero’s Journey story arc is used as a metaphor for REBT, with each stage of the Hero’s Journey related to a part of REBT theory and application. In the current paper, the alignment of the components of the Hero’s Journey and the components of REBT are presented in some detail. The author describes this approach as the rational emotive behavioral monomyth (REBM) and outlines how it can be positioned and applied in practice.

## Introduction

Psychotherapists and practitioner psychologists are often challenged with developing and refining ways to engage clients in change, whether this be in their behavior, emotions, and or their way of thinking about themselves, others, or the world. Client engagement in change processes can be facilitated by developing and maintaining a strong client-practitioner working alliance (Sprenkle and Blow, 2004), but also, through devising communicative devices through which evidence-based strategies can be executed. For example, a cognitive-behavioral approach to psychotherapy affords practitioners a gamut of thought experiments, role playing scenarios, worksheet tasks, practical assignments, and allows for creativity in the therapeutic workspace (see Turner et al., 2023). One of the most common and effective devices is the use of metaphors that encapsulate key elements of a chosen approach (e.g., cognitive restructuring; Muran and Digiuseppe, 1990; DiGiuseppe and Muran, 1992). Metaphors can be used to abstract complex or difficult to portray and understand ideas into allegorical narratives to aid comprehension and provide meaning. Metaphors are non-literal, and can be used to represent a meta truth, or in other words, a metaphor can present an idea in a non-specific way such that the message contained within it can be applied across various specific situations. For example, in the play ‘As You Like It’, William Shakespeare wrote that “All the world’s a stage, And all the men and women merely players, They have their exits and their entrances, And one man in his time plays many parts, His act being seven ages.” Of course, the world is not a literal stage, but by framing it in this way we are encouraged to see the world and our existence within it differently, with the seven ages referring to stages of human life. Life is a fleeting performance, as it were. Thus, a metaphor that concerns a complex idea, or set of ideas, may be more relatable and thus more impactful for and catalytic of change.

As applied to psychotherapy and psychology, a metaphor can serve as a memorable overarching rhetorical depiction of the change process itself. In other words, we can abstract core features of psychotherapeutic approaches to create metaphors with a view to enabling the client to better understand, engage with, and utilize these core features. Stories and analogies can be essential tools in the therapeutic process, because they can provide practitioners with a way to communicate complex psychological ideas to clients (Killick et al., 2016). It is argued within literature that the use of metaphor can be a vital component of cognitive behavioral approaches to psychotherapy (CBTs) (e.g., Blenkiron, 2010; Stoddard and Afari, 2014; Stott et al., 2010), in part because metaphors may act as a bridge between the abstract and the concrete. Creative activities, such as stories and metaphors, can facilitate the explanation of complex ideas relevant to therapeutic change in manner that is potential fun and engaging (Killick et al., 2016).

The current author is obviously not the first to extend the notion of metaphor in psychotherapy, and there are psychotherapeutic approaches in which metaphor plays a crucial role. For example, in Conceptual Metaphor Theory (CMT; Lakoff and Johnson, 1980) metaphor is considered a conceptual tool for structuring, restructuring, and creating reality. Therefore, and as Siegelman (1990) notes, metaphors may be important targets for therapy, in part because metaphors help structure our thoughts and help regulate how we view our present and our past. Malkomsen et al. (2022) argue that metaphors are not uncommon in psychotherapy (e.g., Mathieson et al., 2016), engage human beings emotionally and motivationally in a way that literal language does not (e.g., Citron et al., 2019; Landau et al., 2014), and can mark important therapeutic change through a change in client metaphors (Levitt et al., 2000).

Another prominent example is that of Trial-Based Cognitive Therapy (TBCT; De Oliveira, 2011, 2016), which is based on Beck’s Cognitive Therapy (CT; Beck, 1976) and inspired by the work of Franz Kafka. In Kafka’s novel ‘The Trial’, the lead character ‘Joseph K’ is unaware of the crime he was arrested for and was condemned without having the opportunity to defend himself. In TBCT, de Oliveira proposes that clients embarking upon psychotherapy undertake a similar trial, and tend to accuse and sentence themselves through their negative core beliefs, but are not aware of these self-accusations and are, akin to Joseph K, unable to organize their own self defence (Delavechia et al., 2016). Part of TBCT sees the client address their dysfunctional core beliefs by simulating a courtroom legal trial, where they can role-play as the defendant, the prosecutor, the defense attorney, and also as a member of the jury. TBCT has a unique and distinctive metaphorical approach to the modification of core beliefs, that incorporates a variety of cognitive-behavioral techniques, and has garnered support in extant research for treating conditions such as depression (Hemanny et al., 2020), suicidal ideation (Hemanny et al., 2022), and social anxiety (Caetano et al., 2018).

More broadly, we are story-telling beings who use narratives to communicate on a large scale. We use stories to aid memory, excite attention and emotion, and solve problems. They shape our understanding of the world and our lives (McAdams and McLean, 2013). Stories can shape thinking, promote empathy, and help us exchange information. Story telling is costly, from a time and energy perspective, so our dedication to storytelling over thousands of years is testament to its importance. So, it should not be surprising that in the consulting room, psychologists, psychotherapists, and counselors use stories to aid their practice. The use of stories and parables can be used as metaphors in psychotherapy (Wessler and Wessler, 1980), and can be employed to instigate client paradigm shift (DiGiuseppe and Muran, 1992). One such paradigm shift might include a move from non-autonomous emotion regulation, to autonomous emotional regulation, through a greater harnessing of emotional responsibility. Enabling a client to move from a position of emotional victimhood (i.e., “emotions are something that happen to me and I do not have a say in my emotions”) to emotional responsibility (i.e., “I can shape my emotions, they do not simply happen to me”) is an important part of rational emotive behavioral therapy (REBT; Ellis, 1957).

REBT is based on the central proposition that, on approach and in response to adversity, we have some responsibility for our emotional and behavioral responses, and we can exercise this responsibility through cognitive change (Turner, 2019). In REBT, the extent to which people exhibit functional or dysfunctional emotions and behaviors depends upon the extent to which they hold rational and irrational beliefs. Rational beliefs are flexible, logical, and non-extreme, and are conducive to long-term mental health and goal attainment. In contrast, irrational beliefs are rigid, illogical, extreme, and hinder long-term mental health and goal attainment (Dryden and Branch, 2008). A core feature of REBT is the GABCDE(F) framework (Bernard, 2009; Ellis, 1994; Turner, 2022), which captures the major theoretical components of REBT and also portrays important practical features of REBT.

In brief, the framework is thus:

G – Goals and Values.

A – Adversity (or Activating Event).

B – Beliefs (Rational and Irrational).

C – Consequences of G – A x B that are cognitive, affective, and or behavioral.

D – Disputation (part of cognitive change).

E – Effective new beliefs (part of cognitive change).

F – Functional cognitions, emotions, and behaviors.

**Figure 1:**
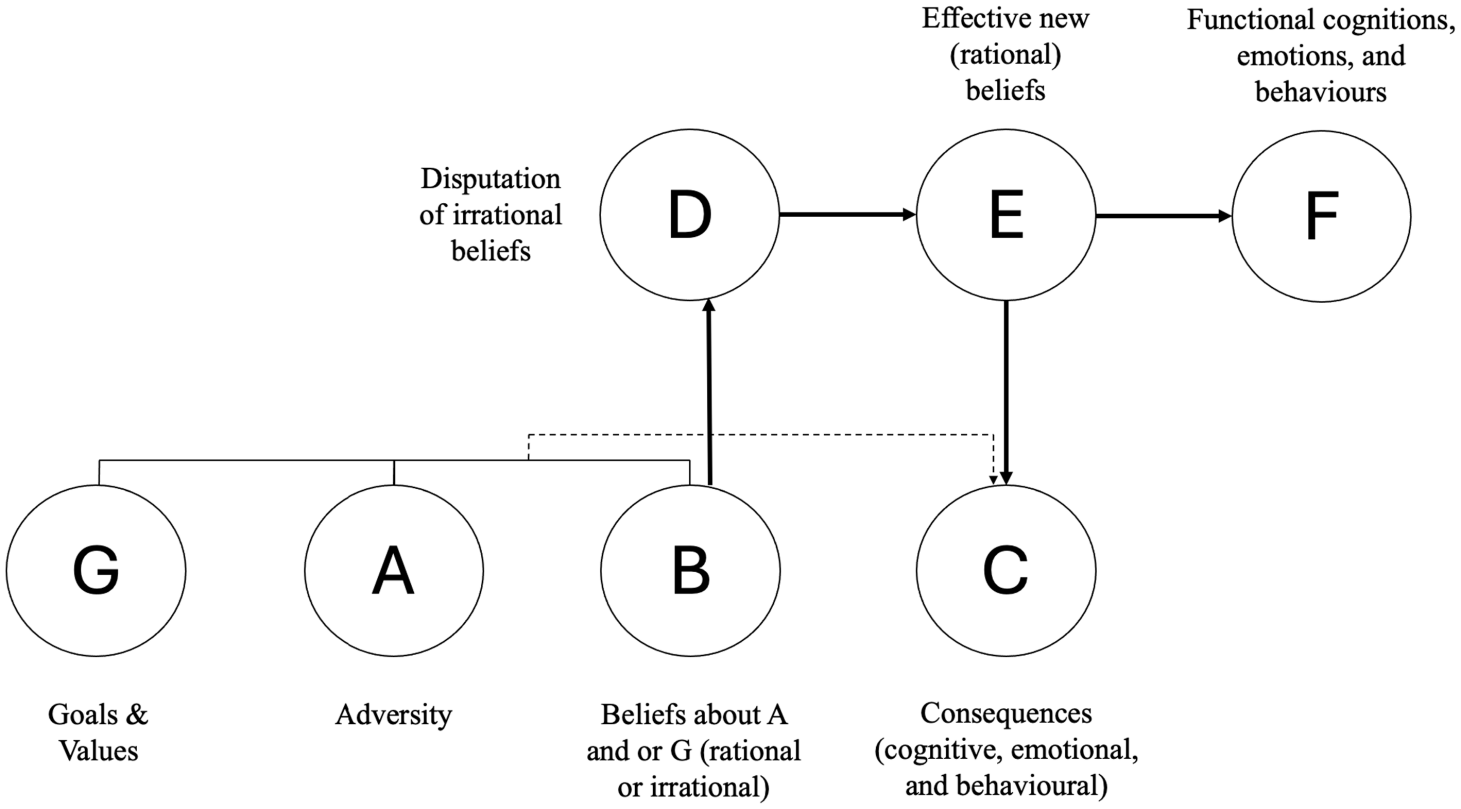
The GABCDE(F) framework of REBT (Bernard, 2009; Ellis, 1994; Turner, 2022). Adapted with permission from Turner (2022).

The theory and practice of REBT is effectively articulated through this GABCDE(F) framework (see also Figure 1). Clients are helped to recognize that when faced with an adversity (A) that is counter to their goals (G), it is their beliefs (B) about the adversity (A), rather than the adversity per se, that governs the functionality of their emotional, cognitive, and behavioral consequences (C). If A is met with irrational beliefs, dysfunctional C’s prevail, while in contrast, if A is met with rational beliefs, functional C’s prevail. Irrational beliefs are disputed (D) following which new effective rational beliefs (E) are instantiated and reinforced, bringing about functional cognitions, emotions, and behaviors (F).

From my first exposure to REBT, I found the GABCDE(F) framework compelling. Why does this GABCDE(F) framework resonate with me so powerfully? Firstly, it is not just me that finds this framework to be compelling. The ABC aspects of the framework have been adopted particularly within second wave CBTs such as REBT and cognitive therapy (CT; Beck, 1976), albeit the specifics are different (REBT focusses more on irrational beliefs, while CT focusses more on automatic thoughts; Young and Turner, 2023). The ABC model is also a motif that runs through emotion science with prominent theories such as Richard Lazarus’ cognitive appraisal theory (Lazarus, 1999), and Gross’ (2014) process model of emotion regulation in which cognitive appraisal and cognitive change are important features. The notion that it is how one interprets and makes sense of the world that is at the center of emotionality, is reflected in ABC, and is echoed in Lazarus’ and Gross’ works. Further, the idea that one can cognitively reappraise (Buhle et al., 2014) restructure or change (Clark, 2014) what one thinks about a stimulus in order to affect emotional outcomes underpins second wave CBTs, as well as Lazarus’ and Gross’ ideas. This cognitive change component is captures by the D and E elements of the GABCDE(F) framework put forth by Ellis (1994). Thus, the GABCDE(F) framework is intuitively appealing, but is underpinned by prominent theory and research (Turner et al., 2021). Clearly, GABCDE(F) framework and the change process offered by REBT places particular importance on cognitive change as a vehicle through which individuals can functionally pursue fulfillment. Cognitive change is an essential emotion regulation strategy (Gross, 2014), and in REBT cognitive change occurs more often than not in relation to irrational beliefs (Turner, 2022).

This misleadingly simple GABCDE(F) framework helps capture the theory and practice of REBT and the more I develop my understanding of REBT the more I appreciate its utility. The current article offers a metaphorical strategy that facilitates the communication of the GABCDE(F) framework and REBT change process to clients, and is something I cover very briefly in my book concerning the use REBT in performance settings (i.e., sport, business; Turner, 2022), namely the Hero’s Journey or ‘Monomyth’ as a broad conceptual metaphor that places the GABCDE(F) framework and REBT change process within a broader notion of human development and growth, which I call the rational emotive behavioral monomyth (REBM).

Put simply, the metaphor is thus: Undertaking REBT is to undertake a Hero’s Journey. Thus, in the current paper, the Hero’s Journey is a metaphor for the change processes taking place when a client and practitioner engage in REBT. I believe one of the reasons that the GABCDE(F) framework has enduring appeal, is in part because the framework is congruent with the enduring Hero’s Journey; the monomythic and archetypal narrative template (Allison and Goethals, 2014; Campbell, 1949), in which a hero embarks on a treacherous adventure and claims victory before returning home—triumphant—but changed. I am not the first person to have evoked the Hero’s Journey as it pertains to psychotherapy. Some authors suggest that the Hero’s Journey can act as a roadmap for change (Williams, 2019), while others see it as a developmental metaphor (Lawson, 2005), focusing on the transformational quality of the Hero’s Journey (Allison et al., 2019). However, I have not seen or read any work, other than my own, that aligns the Hero’s Journey with the core premises of REBT. In the current paper, I argue that the narrative structure of the Hero’s Journey helps us as practitioners better understand and utilize (i.e., communicate and operationalize) the GABCDE(F) framework which at the heart of the REBT change process. Narratives that are recognizable to people, that contain familiar plot lines and themes, are more convincing, understandable, and meaningful (e.g., Rogers et al., 2023). An important feature of the Hero’s Journey is a change in the consciousness of the hero, such that they experience shifts in ideas, beliefs, and thoughts about themselves and the world, which is a feature shared by REBT. But one of the potential problems with the use of metaphor is the assumption of shared (client-practitioner) meaning (DiGiuseppe and Muran, 1992). I posit that using the Hero’s Journey as a metaphor can assuage this issue somewhat due to its implicit appeal to clients, because it is an almost universal narrative pattern that transcends many cultural boundaries. The Hero’s Journey is ubiquitous in story telling (including books, movies, video games). It is likely that the client’s favorite film or story follows the Hero’s Journey, at least in part.

The Hero’s Journey underpins a great many narrative works including films, books, video games, and can be used to make sense of our own trials and tribulations in life.

**Figure 2:**
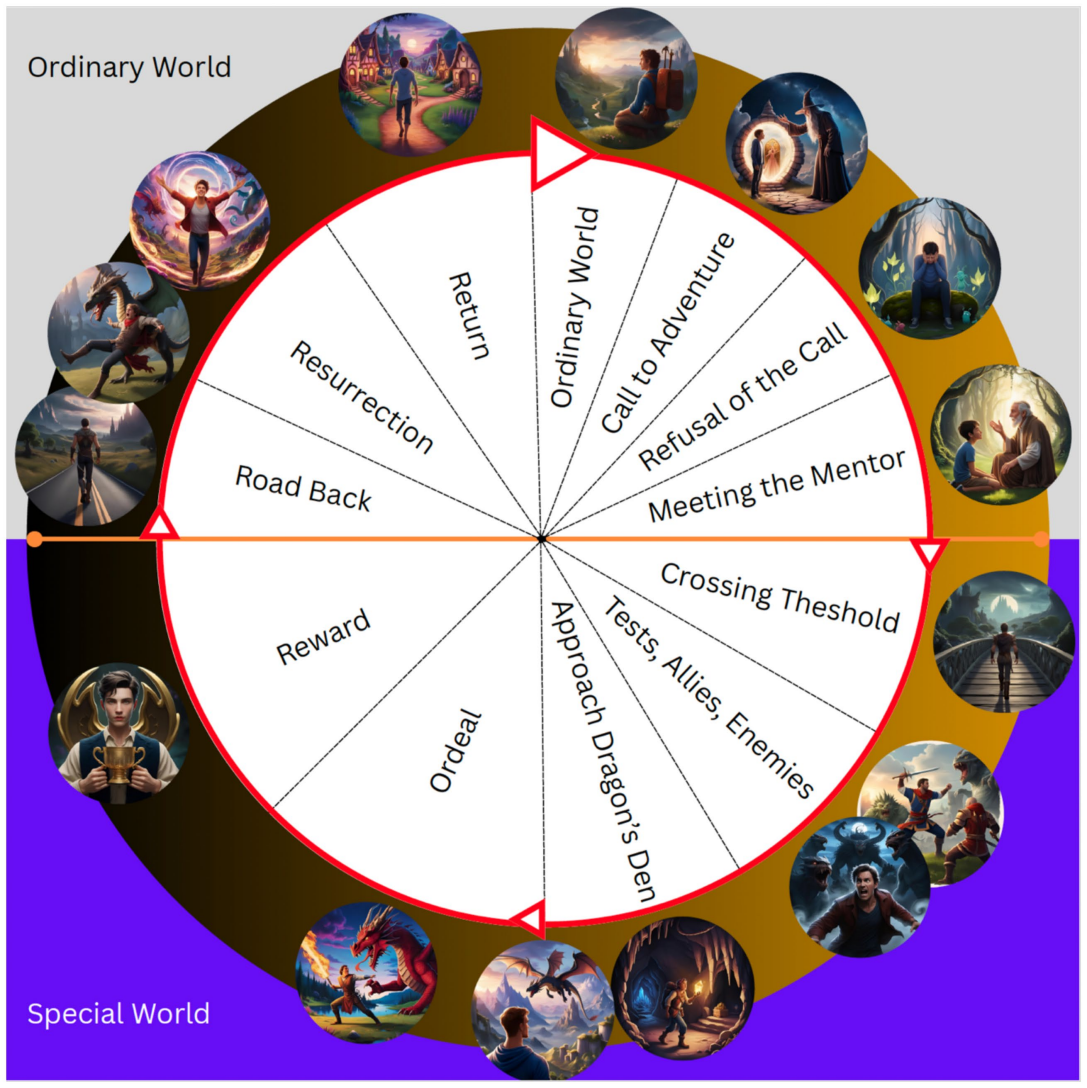
Illustration of the Hero’s Journey using the stages outlined in *Winning the Story Wars* (Sachs, 2012, p. 148). Canva’s (2025) generative AI Magic Media tool was used to create each separate image using the prompt structure; “human *ENTER HERO’S JOURNEY STAGE* in fantasy world.” For example, for the ‘meeting the mentor’ stage, the prompt “human meeting mentor in a fantasy world”.

In Figure 2, you can see an illustration of the Hero’s Journey that has been inspired by Sachs’ (2012) portrayal, and by the work of Christopher Vogler (2020), but was first elaborated by Joseph Campbell (2008) as a hero-quest. Sachs’ and Vogler’s portrayal of the Hero’s Journey differs from Campbell’s original portrayal by simplifying the monomyth into fewer stages, making it more accessible and applicable across domains such as film, writing, and advertising. Indeed, Sachs’ interpretation of Campbell’s original work serves to cast the Hero’s Journey as a way to engage audiences, and as such, fits the purpose in the current paper to engage clients in REBT.

The psychological significance of the monomyth was a key part of Campbell’s work, informed most notably by Carl Jung. The Hero’s Journey can be seen a metaphor or archetype for the inner adventure Jung described as individuation, or the becoming of the self (whole) (Jung, 1959). Archetypes are universal and heritable symbols and themes in the human psyche, thus explaining in part the innate appeal and resonance of the Hero’s Journey. Jung thought that the narrative archetypes observed throughout history exist and operate in the deeper parts of our unconscious, especially in the heritable layer he called the collective unconscious (Jung, 1959, 1967), an idea which dates back to Jungian writings as early as 1902. For Jung, the collective unconscious contains evolved and universal cognitive structures that manifest as archetypes seen in a panoply of myths cross-culturally. Joseph Campbell aligned his thinking with Jung, and thought that we identify, understand, and empathize with archetypes because they symbolize our evolutionary past. Based on Jung’s (1959, 1967) work, the Hero’s Journey is perhaps a shared inherited and primordial symbolistic narrative that captures our evolutionary journey, prevailing through unimaginable danger and pain, from our primitive ancestorial existence to our less primitive present state. Thus, for Jung and Campbell, the Hero’s Journey is a profound externalized allegorical representation of a universal internal (conscious and unconscious) journey (or psychic process) through life toward individuation. Individuation is to become someone undivided, at peace with themselves, aware of their feelings, emotions and character traits, being a functioning member of social structures, and knowing what they want from life (Bassil-Morozow, 2018).

Aside from the psychology and psychotherapy domains, the Hero’s Journey has been influential in many ways across various other domains, most notably in marketing and in cinema. Rather than me lay out all of the examples here—take a look at the illustration and map it against your favorite films. It fits my favorite film—The Matrix (do not get me started on whether Neo or Agent Smith is actually “The One”). But I do not want to dwell on that here—my aim is not to provide a full review of the Hero’s Journey and its influence, as this would be tangential to my purpose here, which is to align the Hero’s Journey to the use of the GABCDE(F) framework as part of REBT. Suffice to say, the Hero’s Journey is deeply imbedded within many cultures and our psyche, reflecting an archetypal narrative that has become somewhat of a cliché in story telling—in part because it is appealing, powerful, scalable, and marketable. This is, I believe, partly why the GABCDE(F) framework is compelling and useful, because it aligns with the way we have been making sense of the world through stories and narratives for a very long time.

The REBM proposed in the current paper represents an articulated alignment between REBT and the Hero’s Journey which differs from previous metaphor-driven approaches to psychotherapy. While CMT (Lakoff and Johnson, 1980) provides a viable evidence-based logic as to why the REBM might be a useful approach to psychotherapy, it does not specify a Hero’s Journey (or any particular) metaphor. There is of course clear theoretical lineage between CMT and REBM, not least because REBM makes use of the CMT notion that one can understanding one domain (e.g., emotion regulation) in terms of another (a Hero’s Journey) (Kövecses, 2020). But the REBM provides a specific thematic structure to the ‘mapping’ of the psychological domain on to the mythical domain. The TBCT uses metaphor in different and specific, but no less meaningful or valuable, ways than the REBM, and is garnering strong evidence for its effectiveness. The specific metaphor used in TBCT is that of a courtroom trial, whereas in the REBM a less specific metaphor is used. The Hero’s Journey is not a specific story or path, but reflects an archetypal narrative structure within which many stories can be told. In the REBM it is the change processes occurring during REBT that specifies the application of the Hero’s Journey in the therapeutic context. As such, the author takes inspiration from TBCT, but offers a novel approach to REBT that differs conceptually and in its application from TBCT.

Before I move onto exploring the GABCDE(F) framework within the metaphorical context of the Hero’s Journey, I just want to be clear that I am not the hero in my GABCDE(F) analogy. Just in case you thought you were about to read a self-adulating ego-massage (!). The client is the hero. Bassil-Morozow (2018) in their Jungian toolkit for storytellers suggests that the hero “symbolizes the individuating, progressing human being whose everyday struggle with life and reality is metaphorically presented in the form of superhuman tasks such as dragon-slaying…” (p. 33). In REBT we are not expressly concerned with individuation, but we are trying to support our client-hero with their struggles and reality. Further, the client-hero while not attempting super-human feats, are attempting to achieve something they may have thought they could not (e.g., self-regulation, belief/emotion/behavior change). Thus, painting the client as the hero is appropriate as it pertains to the metaphor of REBT as a Hero’s Journey. Indeed, for many clients, everyday life can be a struggle, and it may help them to characterize their endeavors in a ‘heroic’ narrative (Bassil-Morozow, 2018). As the hero-client moved toward rationality with emotional insight (a chief aim of REBT), they drive their story toward psychic perfection with sage-like rationality, an ideal that is impossible to reach but worth aiming for. Our role as practitioners will become clear later in this piece, but this role is not what I would consider to be ‘heroic’. Of course, a practitioner can embark on their own Hero’s Journey (as all human beings can)—but here I place the client as the central protagonist of the REBM. In articulating the structure and individual elements of the Hero’s Journey, I draw mainly on Vogler’s (2020) work.

## The Hero’s Journey and the GABCDE(F) framework

**Figure 3:**
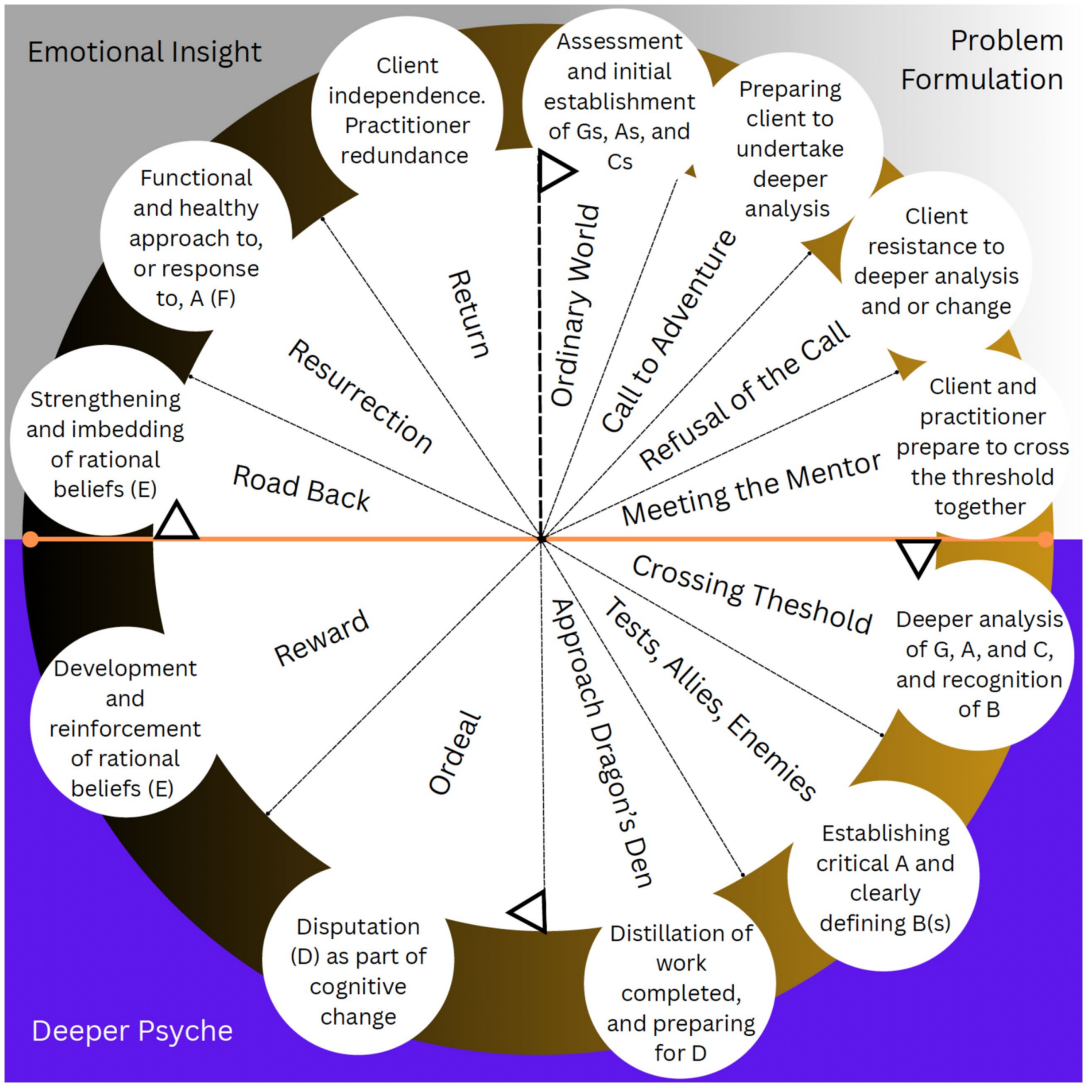
Mapping of the Hero’s Journey from Figure 2, to the aspects of REBT covered in the current paper. Canva’s (2025) design tool was used to create this diagram.

I position the GABCDE(F) framework within the Hero’s Journey (monomyth), i.e., the REBM, in order to illustrate the work done collaboratively by the practitioner and the client. It is the client’s journey ultimately—the practitioner for sure facilitates this journey and acts as a guide (mentor) to the client (Hero). Our role is to aid the client in their personal quest. Think Obi-Wan Kenobi in Star Wars, or Morpheus in The Matrix. Indeed, as is typical in REBT, the client does most of the work and the practitioner uses time spent together to help the client to stay on course. Next, I take each aspect of the Hero’s Journey and align it with REBT, and specifically, the GABCDE(F) framework, to flesh out the REBM. In Figure 2, I graphically depict the Hero’s Journey as it is typically portrayed, in Table 1 and Figure 3, I briefly align the elements of the Hero’s Journey with the GABCDE(F) framework of REBT, offering details from the typical (not REBT) Hero’s Journey for quick and easy comparison to the REBM. In what follows, I cover each element of the REBM in detail.

**Table 1:** Details for each element of the Hero’s Journey (monomyth), the alignment between the REBT GABCDE(F) framework and the monomyth, and the indicative session in which each element takes place.

Stage of monomyth	Key story elements	Session number	Related aspects of the REBT GABCDE(F) framework
Ordinary world	Background of the Hero from which they set forth into the Special World. Hero’s inner and outer problems are already present in the Ordinary World, and questions arise concerning the Hero’s capacity to, and likelihood of, prevailing through self-development and growth. Often, something is revealed to be missing from the Hero’s character—skill, knowledge, or a character trait—that needs to be fulfilled in order to assuage these inner and outer problems. The Hero is sometimes seen to be suffering in some way and there appears to be something at stake if the Hero does not prevail.	1	Assessment and initial establishment of Gs, As, and Cs.
Call to adventure	Gets the story moving and provides the impetus for the Hero to step outside of the Ordinary World which is beset by existential danger into an uncertain by necessary path of adventure. The Herald typically calls the Hero to adventure, and this role is sometimes incorporated into The Mentor who has the Hero’s best interests in mind. The Call to Adventure can be unsettling and uncomfortable for the Hero, but is necessary for the commencement of the adventure. There can be more than one Call to Adventure, often needed for a reluctant Hero.	2	Preparing client to undertake deeper analysis.
Refusal of the call	Often manifested in the Hero being in denial of the issue present in their Ordinary World, and the Hero can be temporarily hesitant, which is understandable given what is at stake and the unknown danger that awaits the Hero if they accept the Call to Adventure. The Hero may initially consider, and even attempt, to avoid the adventure in the interest of preserving their life with excuses and facile justifications. But importantly, persistent refusal and a desire and effort to remain within the Ordinary is disastrous for the Hero.	2	Client resistance to deeper analysis and or change
Meeting the mentor	The Mentor can be embodied by a character in the story or can represent the inner and higher self (e.g., the conscience) that orients the Hero toward the highest aspirations. The Mentor is a parent-like figure that offers the map for the Journey, and or vital information needed for the voyage, as well as offering motivation, energy, guidance, training, and gifts (such as tools and knowledge) to the Hero to facilitate their journey.	3	Client and practitioner prepare to cross the threshold together.
Crossing the threshold	This is usually where the adventure really begins and there is no turning back. There is normally a shift in energy when the threshold is crossed, representing passing into the ‘special world’, with the Hero demonstrating their willingness and commitment to take on the unknown. This willingness if fueled in part by high stakes—the consequences of not crossing the threshold are worse than if the threshold is cross—but also the Hero is often encouraged to cross the threshold by an external force. The threshold is often depicted as a river, wall, gate, or canyon, for example, and there is often a Guardian of the Threshold who blocks the way. This Guardian can be an illusion, but after acknowledging the Guardian, the Hero must figure a way through or past the Guardian to continue the journey.	3	Deeper analysis of G, A, and C, and recognition of B. Psychoeducation in the GABC aspects of the framework.
Tests, allies, and enemies	Tests provide trials, difficult obstacles, traps, and challenges that prepare the Hero for greater future Ordeals. In these Tests, the price of mistakes is high, but not life or death. There is danger and excitement, and the Mentor continues to train the Hero through these Tests with new skills and ideas. The Hero at this stage must also figure out who their Allies and Enemies are. Allies are companions and partners, and often reflect the Hero’s conscience—internally can represent powerful internal forces that come to the Hero’s aid. Allies advise, warn, and challenge, and can appear as a guardian spirit, but can be represented by the Mentor too. This is important because the Hero will encounter Enemies, or ‘the Shadow’, often depicted as villains and assailants (monsters, aliens etc). The Shadow cab also reflect the suppressed energy of the dark side, a monster of the inner world. Internally, this can be represented by things the Hero does not like about themselves, or qualities they have tried to eradicate but still lurk within. The Hero is in constant battle with the Shadow. The Mentor can wear the mask of the Shadow at times, in order to challenge and provoke the Hero. The Hero must bring into light and defeat the Shadow to prevail, and ultimately, this phase is all about the Hero accumulating power and knowledge for entry into the innermost cave (where the Ordeal takes place).	3	Establishing critical A and clearly defining B(s).
Approach the Dragon’s Den	This stage is about approaching the heart of the Special World where the Hero makes the final assault. The Hero plans, organizes, and is fortifies for the Ordeal, full of confidence. The Hero must stay alert and pay attention to what they have learned so far and the lessons that their preceding journey has provided them. Fortification is needed because the Hero will not be able to just go into the cave and take the Treasure the seek—the ruler of the cave will not simply capitulate and will defend the Treasure ferociously. The Hero must prepare for complications. The Mentor helps the Hero to rededicate to the Journey and instill some urgency into their pursuit.	4	Distillation of work completed, and preparing for D.
Ordeal	Takes place in the deepest chamber of the inner most cave. This is symbolic for the deepest point of the Hero’s self, and can represent the Hero’s fears and insecurities, which are demonized. The Ordeal represents the Hero’s greatest challenge and as such, they face their most feared opponent—facing The Shadow, battling the ultimate opposing and deadly force. During the Ordeal the Hero often dies, which can be literal or symbolic, but survives this death. The Hero returns from this death changed. During The Ordeal, the opponent is defeated, either destroyed or forced to retreat, but not easily—it is labored, difficult, meaningful. The mentor is often witness to this great battle. The journey so far has all been leading up to this, and nothing will be the same after The Ordeal. However, The Ordeal is not the climax of the Hero’s Journey, but is for sure the ‘main event’.	4	D as part of cognitive change.
Reward	After the The Shadow is slain or vanquished, the Hero forthrightly takes possession of what they have been seeking. The Hero’s safety has been transacted for this reward, which can be something physical like gold or a sacred sword, or can be knowledge or wisdom. The Hero is often imbued with new powers, better perceptions, and an ability to see through deception. With new possibilities now opened up, the reflects on the battle and recounts what they have learned. There is a moment of clarity as the Hero is now able to perceive reality, but also a self-realization and deeper insight into who they truly are and how foolish they may have been up to this point. There is a shift in behavior too, with the Hero appearing more mature and wiser. This is useful, because there are more Ordeals ahead.	5	Development and reinforcement of rational beliefs (E).
Road Back	The Hero has to make a decision to stay in the Special World or to return to the Ordinary World. The Hero may have found comfort in the Special World, but they tend to choose home. If the Hero is to make it back home in order to share the Reward with those in the Ordinary World (which is an important purpose of the Journey), they must undergo further trials and tribulations, implementing what they have learned on their journey. The Shadow may re-emerge and retaliate with great strength, or a renewed danger may arise to hunt down the Hero and hinder their journey back and attempt to wrestle the Reward from the Hero. Alternatively, the Hero may give chase to the escaped foe from The Ordeal, who has become more dangerous and presents an existential threat. These physical challenges can be symbolic of personal psychological struggle—internal issues that have been challenged may retreat for a time but arise powerfully, striking back when threatened. The Hero can experience a reversal of fortunes and face sets backs, and catastrophe, but the motivation to persist along the Road Back is recapitulated.	6	Strengthening and imbedding of rational beliefs (E).
Resurrection	The hero faces an additional moment of death and rebirth, and usually functions as the last and most dangerous and significant challenge or test. Does the hero retain what they have learned? Will they in their old ways, or in their new transformed way? Can they apply it to new challenges? Can they take action and be decisive in this momentous trial? In the Matrix, Neo narrowly escapes with his life after facing Agent Smith for the first time, but then faces and defeats Smith in the final battle, but only after being resurrected by Trinity. Neo attains a higher awareness in this cathartic climax. The hero experiences a final purification, a cleansing, a final change, before entering back into the ordinary world. The hero typically sheds the character they have developed over the journey and builds a new one in order to return home. But the hero must sacrifice something in order to be fully resurrected; an old habit, an old belief, for example.	6	Functional and healthy approach to, or response to, A (F).
Return	The hero has survived, is meaningfully changed, and returns to the ordinary world to share their earned wisdom (and or physical treasure) with others. The hero acts in their daily life in the way that has been solidified by their journey and used the lessons of the journey to help others. For example, Frodo returns to the Shire, changed forever. They are different and they see things differently. Importantly in The Return, the hero brings with them an Elixir that has been hard earned on their journey. This Elixir can be something of physical value like a cure, gold, or an ancient prize, but is often figuratively described, such as knowledge, wisdom, a secret. It is proof that learning has taken place, and brings the hero and those around them greater awareness.	7	Client independence. Practitioner redundance.

It is assumed that each session is 45–60-min in length.

### Ordinary world

In practicing REBT, the early phases of my work with the client are characterized by establishing what the client’s “Ordinary World” is like. What are their contextual goals and values (G)? We do not superficially establish Gs, we try to get to the deeper, more humanistic, roots of the client’s goals. What drives G? What do they desire? What does fulfillment (of potential) look like to the client? Where is the congruence and incongruence between what they want, and the reality? What is stopping them from getting what they want? What emotional and behavioral consequences (C) are they exhibiting in relation to the supposed activating event (A) that is precluding goal attainment? In what ways is the client suffering, and how might they be contributing to their own suffering? What happens if the client does not address their suffering? What emotional and behavioral consequences have they noticed, or do they expect to experience, in light of thwarted Gs and preponderant As. What do they stand to gain and lose from confronting their issues? How would they like their ordinary world to change, and who would they like to become in this new world vision?

All this talk of ‘vision’ might all sound very lofty, but at its core and in REBT parlance, I am assessing and establishing the clients Gs, As and Cs. G is fundamental to the REBT work, and self-regulation more broadly, because it is by having goals that we bring about adversity, and it is in relation to goal pursuit that we experience positive and negative emotion (Turner, 2022). As I move toward a goal, positive emotion is elicited, and as I move away from a goal (i.e., goal is or might be thwarted), negative emotion is elicited (Carver and Scheier, 1998). So, I am assessing the nature of A, in what ways A inhibits G (as perceived by the client), and what feelings and actions are evinced as a result (C). I am carefully assessing C to understand whether and to what extent the client’s emotional and behavioral reactions to A are currently (or likely to be) healthy (negatively valanced, but functional) or unhealthy (negatively valanced, but dysfunctional). This is to be understood within the context of the client’s world (how they perceive it), not mine. At this stage, I am relatively passive (I listen more than I speak), applying active listening in order to help the client to go deeper into their goals (G), emotional issues (C) and the supposed events that precede them (A). I am interested in assessing all of this in a literal or ‘lived’ sense (i.e., experienced As and Cs), and also in a hypothetical or imagined/predicted sense (i.e., could occur, given the specific Gs). Put differently, in the context of the client’s Gs, what As and Cs have occurred, could occur, or will occur, in pursuit of those Gs?

Aside from a GAC assessment, in this ‘Ordinary World’ stage I am also gaining an understanding of the client’s current strengths and areas for development, so that I can position the next phase (Call to Adventure) appropriately—I do not want to call the client to an adventure for which there are significant contraindications. Also, I want to reveal the client’s skill and knowledge deficits so that I can plan their skills and knowledge development. In the traditional Hero’s Journey, the Hero has many strengths and many weaknesses, and the articulation of these is important for grounding the character. In REBT, I want to understand what the client currently can and cannot do in terms of meaningful pursuit of their goals, so that we can approach the work with these attributes in mind. In sum, together with the client, I am trying to gain a detailed understanding of how the client came to be before me today, and how best we can move forward.

### Call to adventure

In REBT, the Call to Adventure is evoked in at least two ways. First, the client has usually in some way been driven to seek our help, often by a problem in their lives (e.g., an A or a C) or a desire that is unfulfilled (e.g., a G that they want to attain). This is what often gets the client into the room with me. They have been compelled in some way, either intrinsically or extrinsically, to seek some support from a psychologist/psychotherapist. But it is the second evocation of the Call to Adventure I focus on here, which is the instigation and initiation of the change process that takes place once *within* REBT. In this second evocation, I the practitioner initiate the Call with the work that has already commenced (i.e., assessment is taking place). Just like for the Hero in the traditional Hero’s Journey, for the client this instigation can be unsettling. In being called to adventure, often The Hero is unaware that there is an issue within their Ordinary World and may have getting by using a range of (maladaptive) coping mechanisms (e.g., avoidance, withdrawal, substance misuse). In other words, the client at this point may not understand that their current state of being (their values, goals, perceptions, beliefs, emotions) is interrupting their wellbeing. They are in the room with me because something is amiss, they are suffering, and or they are not moving toward their goals. But they may not be aware of how their current orientation in the world may be part of what is thwarting their goal pursuit.

Thus, my call to adventure to the client is this: Are you prepared to go deeper and figure out what is really going on here? In REBT terms, are you ready and willing to explore the role your ideas about yourself, others, and the world, might be playing in your malaise? Do I have your permission to interrogate your deeply held schemas and philosophies in order to help you on your journey to fulfillment? Are you prepared to take on this adventure into the deeper parts of the self, to uncover the things that might be holding you back, and discover ways in which you could grow and develop? I am also trying to help the client to understand which of their coping mechanisms are maladaptive for goal attainment, helping to motivate the client toward a solution. In the traditional Hero’s Journey this is sometimes portrayed as the Hero feeling like they have run out of options because what they are currently doing is not working anymore, or is making things worse. And in my experience, the client is often making things worse for themselves. To clarity, for sure many clients are facing uncontrollable adversity and are understandably suffering under the weight of a struggle. Life is hard, and we suffer by nature of being human. But often, they are also contributing to their own suffering in ways that are changeable. So, often, clients have been applying ineffective or maladaptive coping strategies to life’s adversities, and at this point, can be forgiven for feeling a little helpless.

Having established the client’s Gs, As, and Cs, and undertaken as good an assessment as I can (assessment is ongoing technically), I as the practitioner instigate the Call to Adventure by presenting the client with my understanding of their presenting issue. You could think of the practitioner at this point as The Herald, an archetype often embodied by the Mentor in the traditional Hero’s Journey. Usually, I will package the information that the client has provided to me, or that we have explored together, thus far within the G, A, and C aspects of the framework. It is important to get this right, and to check my assumptions with the client so that we can move forward forthrightly, under no false assumptions. In brief, I am helping the client to see that their unhealthy Cs could be emerging as a consequence of two related occurrences: (a) the experience of an adversity (A—perceived or imagined) that is incongruent with their goals and values (G), and (b) the holding of, and engagement in, maladaptive beliefs (B) concerning A, or G or the incongruence between G and A. The former (a) is sufficient for emotional reactivity per se (see the work of Richard Lazarus). That is, evidence indicates that having a meaningful goal thwarted is grounds for negative emotionality (Kreibig et al., 2010). The latter (b) is sufficient for this emotional reactivity to become unhealthy or dysfunctional (see the work of Albert Ellis). This is an important piece of the story, because we are laying the groundwork for exploring B as part of the GABC framework, which is key aspect of specific REBT (Ellis, 1977). In the Call to Adventure, we are inviting the client to take a deeper dive to uncover potential beliefs that are, in part, driving and exacerbating their suffering.

### Refusal of the call

In practicing REBT, it must be recognized that deep belief change is not easy for most clients, or indeed for the practitioner. Indeed, REBT writings and training illustrate the complications of deep belief (philosophical) change (e.g., Jones and Turner, 2022). In REBT research literature we often guilty of presenting REBT too simply—we perhaps prioritize scientific methodological content in research papers over and above the practical details (see Wood et al., 2020, for an exception). But imagine holding a belief since as far back as you can remember, and then realizing that not only might this belief be contributing to your suffering, but also that the reason for this is because the well-rehearsed and entrenched belief is bereft of truth and logic. I do not use this forceful language with clients, but I do sensitively deliver this revelation (if they do not realize it for themselves) in a manner that befits the particular therapeutic relationship that has been established (and is still being developed). Not only is the client probably self-perpetuating some of their issues, they also within them hold the only solution that must be excavated and utilized if they are to overcome their issues. Only through taking a dangerous journey can the Hero prevail. Only through undertaking the difficult work of therapy can the client prevail. It is no wonder that often, the client refuses the call to adventure.

In my experience, clients express their Refusal of the Call in four main ways: (a) they resist the notion that they are in part responsible for their emotional suffering (“it is nothing to do with me, *they* are the ones who are treating me so badly”), (b) they resist the notion that they could possibly hold beliefs that do not meet high standards of truth and logic (“are you saying that I am stupid?,” (c) they display discomfort in response to the idea of being challenged about their beliefs and seek to avoid confrontation (“cannot you just help me *feel* better in the moment without questioning the content of my beliefs?”), and or d) they supply a litany of reasons that prohibit them from undertaking the work required as part of REBT.

This reluctance must be addressed, or else the journey either stops here, or continues on a path to nowhere, or toward an inelegant solution that superficially addresses the client’s issues, or worse toward further and worse suffering. In the traditional Hero’s Journey, the Hero who continually refuses the Call to Adventure is met with tragedy. In REBT, unless the client is able to take responsibility for their emotions, is able to understand that some of their thinking could be faulty, is able to tolerate some challenge to their thinking, and is willing to undertake the necessary work, then it is probably not possible to execute successful REBT. But most importantly, the practitioner should recognize that a Refusal of the Call by the client, or some resistance to change, is normal and should be addressed with the client just like anything else in the work. Afterall, is it possible that this resistance is in itself an indicator of maladaptive beliefs concerning change, emotional responsibility, competence, or discomfort (e.g., People absolutely should not challenge my way of thinking, and I cannot tolerate having to make changes in my life”). In sum, for various reasons the client may demonstrate some resistance to the work, which should be viewed as normal and addressed forthrightly to assuage it. In the aforementioned scene from The Matrix, Trinity stops Neo getting out of the car and says “Please Neo, you have to trust me…Because you have been down there, Neo. You know that road. You know exactly where it ends. And I know that’s not where you want to be” Neo gets back into the car. We as practitioners must encourage the client to understand the perils of chronic resistance and motivate them to move beyond the safe and certain confines of their Ordinary World.

### Meeting the mentor

In implementing REBT, as the Mentor, I have already initiated the Call to Adventure and I have encouraged and motivated the client to move past their resistance and reservations (Refusal of the Call). So, Meeting The Mentor is about a change in my position as practitioner, rather than the client meeting me for the first time. Up to now, I as the practitioner have taken on the role predominantly of empathic listener, and when calling the client to adventure, I am starting to take the role of mentor. An alliance is formed (“let us go on this journey together”) and we cross the threshold as a collaborative team in the next stage of the journey. As I have outlined elsewhere (Turner and Barker, 2014), my early interactions with clients are largely person-centered as I gather information and understand the client’s goals, values, motives. However, as my role changes to “Mentor” I can be more active-directive in my engagement with the client, and can advise and guide, in line with REBT practice (Turner, 2022). Part of the Mentor’s function in the traditional Hero’s Journey is gift giving, which is often portrayed as the giving of tools and knowledge useful at key parts or the journey ahead. Think of Obi Wan Kenobi giving Luke Skywalker his father’s lightsaber. In REBT, this can take the form of psychoeducation concerning the GABCDE(F) framework itself, but also the skills of introspection (or internal enquiry), disputation (critical thoughts), and range of psychological skills (e.g., self-talk, imagery). Gifts should be earned by the Hero, and the client can earn these gifts by committing to the work we are doing in session and out of session, engaging in deep introspection, homework, and the change process per se.

The role of Mentor in REBT is not an authoritative position. I am the client’s aid—they are in charge as autonomous sentient human beings—I am offering advice, support, and guidance. Indeed, we can learn a lot from clients and there can be various role reversals. As Albert Ellis was developing his theory (which became REBT), he obtained some brilliant revelations from his clients (see Ellis, 1994). Given the likelihood of resistance (i.e., Refusal of the Call), our job here is to support the client in understanding that, for meaningful change to take place, they would benefit from crossing the threshold. This means potential challenge for them, potential discomfort, potential emotional pain, as we excavate and dispute deeply held ideas. We can encourage the client to understand that we do this excavation and disputation only to help them to discover the genesis of their issues, and to find solutions at the root of the issues rather than at the periphery. In this way, we mentor the client and prepare them to cross the threshold, into the unknown (for us both). Over the course of the work, just like in the traditional Hero’s Journey, we encourage the client to become their own Mentor, assimilating the Mentor into their character as a representative of their higher self and an inner code of behavior. In REBT, we seek to make ourselves as practitioners redundant.

### Crossing the threshold

In REBT, as I indicated in the previous stage, the client and I cross the threshold together. The crossing of the threshold is required in order to undertake a much deeper and more rigorous assessment of G, A and C, and the assessment for the presence and nature of B, which will take place fully and very specifically in the next phase (Tests, Allies, and Enemies). In the Hero’s Journey, this means passage into the ‘special world’, and in REBT this special world is certainly the deeper psychological world that resides underneath immediate conscious awareness. This represents entering into the unknown, and therefore can be met with some apprehension from the client. Just like in the Hero’s Journey, the practitioner (Mentor) prepares the Hero for this passage and in particular facilitates the Hero through any apprehensions they may have. As mentioned, a typical archetype found in the Hero’s Journey is the Guardian of the Threshold, which in my REBT analogy I consider to be the danger that is perceived and the anxiety that is often experienced by the client regarding the prospect of going deeper into their psyche—into the unknown. We as the practitioner can help the client to acknowledge this barrier, and help them to move past it by helping them to consider the stakes at play. What are the consequences of not crossing the threshold? In other words, what is to be gained and lost by decided not to undertake this deeper analysis, even it brings with it some discomfort? We can also help them to self-efficaciously reflect on their capacity to undertake this deeper exploration by drawing on times they have faced the unknown and prevailed.

Crossing the threshold can only happen effectively if the client is willing and able to introspect, and bring the contents of that introspection into the session by verbalizing their thoughts and beliefs. If the client trusts us to guide them, we can take them deeper and deeper into their psyche—this is necessary in order to uncover core beliefs at the center of their issues. The rapport we have with the client is important. We cannot force this introspection, but we can provide the forum, space, and structure to help the client to do this. As Morpheus says to Neo in The Matrix, “I can only show you the door. You’re the one that has to walk through it.” Why is this depth necessary? In part, because irrational beliefs are not just waiting around at the forefront of the client’s mind, ready to be discovered and challenged. It usually takes a bit of digging to get access to these deeply held core beliefs. So here, as we cross the threshold, we get the client used to the idea that there are ideas lurking under the surface that can shape how they think, feel and behave when faced with As. We can educate the client in the GABC framework and show them the door they will be walking through as we proceed with our work. Crossing the threshold is about preparing the client to undertake a deeper assessment of their issues.

### Tests, allies, and enemies

In implementing REBT, when the threshold has been crossed, we put into action the aforementioned introspection. We can use Socratic questioning, or techniques like inference chaining, to facilitate a downward (or more inward) exploration into what is at the core of the client’s issues. We can determine whether the emotions at C are healthy (adaptive) or unhealthy (maladaptive). We can test the client’s inferences and challenge the client to be open and honest with what has contributed to their issues even though it might be uncomfortable (Tests), we can help them to uncover deeply help irrational beliefs at the root of their suffering (Enemies), and also help them to identify beliefs that are helpful to their goal attainment, and personal strengths they hold that could help them in their journey (Allies). We begin to understand the precise adversities (A) to client goals (G) that are triggering their irrational beliefs (B); we call this the critical A (Dryden, 2009). We gain a detailed understanding of the precise ways in which this G, A and B connection are underpinning unhealthy emotional consequences (C) and how these consequences are expressed (i.e., in behavior).

Here my aim is to help the client arrive at a critical A—an inference that is at the heart of the emotional reactivity. For example, the client might say “I have been dropped from the work team,” but on further exploration it is the manner in which they were dropped that is the real issue (“the manager was disrespectful”), and even further, what this treatment from the manager means to the client’s perceived value (“the manager does not value me as a person”). This gets us closer to B—we can ask the client what it means to them to not be valued by the manager, we can ask the client what they are telling themselves about the manager not valuing them. We might, together, realize that the client demands to be valued, that they see their perceived undervaluation as terrible, and that they view themselves as ‘a useless loser’ due to this undervaluing. They may even believe that the manager is a bad person. We can ask the client to what extent they believe that what they are telling themselves about this situation is leading to helpful or unhelpful consequences (C). In this stage, the client may experience a revelation—that while the event they experienced was bad, unwanted, and to some extent conducive to emotional suffering, their beliefs about the situation have exacerbated their suffering and are contributing to this prolonged state of malaise. Through engaging with these Tests (frank, challenging, and uncomfortable conversations), Enemies (irrational beliefs), and Allies (rational beliefs and personal strengths) the client adds meat to the bones of the GABC aspects of the framework, gaining a deeper and broader understanding of their issues and the contributing factors. The practitioner is helping the client to illuminate the critical A and irrational beliefs, just as the Mentor in the Hero’s Journey helps the Hero to illuminate the Shadow. By doing this, the key determinants of the client’s issues can be approached accurately and confidently.

### Approach the Dragon’s Den (or the inner most cave)

In REBT, at this stage, the client and I would distill the work we have done so far. We would take stock, and clearly arrive at and explicitly articulate a critical ‘A’, at least one B, and at least one C (with its cognitive, emotional, and behavioral features articulated). We may even commit this information to paper, as I often do with clients. What should become clear to the client is that B is at the center of their unnecessary suffering, and thus, we should approach B and do something about it. In my example, the Dragon occupying the Dragon’s Den is the B; the irrational belief. What is it about irrational beliefs that makes them Dragon-like? Well, we do not have full control over our irrational beliefs. They reflect personal mythologies (“I must succeed!”), age-old malevolent tyrants, that have been with us for a long time (see Turner, 2022, for a full discussion on the evolutionary significant of irrational beliefs), that lurk in the depths of our mind. Going back to Jung, he believed that the true Dragon in the Hero’s Journey is the one that exists in our minds, and are inner demons and aspects of ourselves (The Shadow) that we may not want to acknowledge. The Dragon is an appropriate character because it reflects the uncontrollable, the mythical, and the ancient, able to wreak havoc when called into action. We have to go the depths and fight the Dragon (who, in mythology, typically dwells in a cave or underneath a mountain), just like we need to go to the depths of our mind to confront and challenge the irrational beliefs. The Dragon lays dormant, sleeping, like Smaug from The Hobbit. Like the irrational beliefs, the Dragon lays dormant until they are triggered by A, or sought out by pesky psychotherapists and psychologists. The Dragon represents dogma, rigidity, and extremeness (“I must have all the gold, and I could not stand it if it was stolen!”), who likes to deviously twist reality. The Dragon is irrational in their greed for gold, which they cannot use. Lastly, in mythology, Dragon’s usually have one key weakness that needs to be exploited in order to defeat them. The main weakness of irrational beliefs is that they are disputable, quite easily in intellectually terms, by applying empirical, logical, and pragmatic disputational arguments.

In brief, ‘Approaching the Dragon’ reflects the client and I approaching the irrational beliefs that sleeps in the darkened recesses of the mind. As we approach the Dragon’s Den, we prepare the client for an Ordeal; a vital confrontation with the Dragon. In doing this, we make plans, organize, and fortify the Hero to undertake the Ordeal of rigorous disputation of their irrational beliefs.

### The Ordeal

In this stage on REBT, we challenge, argue against, dispute the irrational beliefs—this is ‘D’ in the GABCDE(F) framework. We take on the Dragon. When I say “we” *I really mean* “we.” This disputation (D) process is done collaboratively as both client and practitioner take a critical view of the irrational beliefs, and rigorously (and often vigorously) dispute them. To be clear, we challenge the beliefs, not the individual who holds the beliefs (i.e., the client). We externalize the belief and hold it out in front of us (figuratively and literarily, by writing the beliefs down on paper for example) for scrutiny. So, how do we combat the Dragon (irrational beliefs)? What tools do we need to help us defeat this ominous mythical beast?

In REBT writings, research, and training, authors tend to present three main arguments: empirical (evidential), logical (sensical), and pragmatic (functional). However, there are many other arguments, such as existential, semantic, and paradoxical (DiGiuseppe and Muran, 1992; Turner, 2022). We the practitioner teach these skills to our clients, just like the Mentor furnishes the Hero with useful tools (i.e., weapons, information, resources) that facilitate their journey. But ultimately, we take the irrational belief, and challenge its evidence, logic, and utility (for goal attainment). If the belief is not grounded in evidence, is not consistent with reality, and does not serve an adaptive (or functional) purpose for goal attainment, then we can describe it as irrational and therefore unfit for purpose. Ultimately, and allied to Stephen Pinker’s (2021) conception of irrationality, if the belief is not helping the client to attain their goal, then it is not fit for purpose. Of course, one does not need to use language such as ‘irrational’ with the client—use whatever term captures these beliefs that resonates within the context you are working for the people you are working with.

Jung (1959) states that “the hero’s main feat is to overcome the monster of darkness” (par 284). It is important to note that demonizing the irrational beliefs as ‘monsters’ or by using a Dragon metaphor, can help us to articulate and define the beliefs, externalize them for analysis and disputation, and enable the client to see them more clearly. We project irrational beliefs on to the Dragon in order to dispute them and weaken them. But projections they remain, and the client is made aware that these beliefs are of course internalized ideas that have been developed over a long period of time. As is often the case in the Hero’s Journey, there is a part of the Dragon in the Hero (i.e., The Shadow). For Jung the “Shadow” archetype personifies what we refuse to acknowledge about ourselves that affect us in both direct and indirect ways, such as ‘inferior’ traits of character (Jung, 1959), or in the present case, irrational beliefs. But we must accept the Shadow if we are to develop ourselves and integrate the Shadow into the Self. Thus, in the REBM we are not encouraging the client to eradicate irrational beliefs (which is not possible), but to accept that they have a propensity to hold irrational beliefs and to develop methods to counter these irrationalities. We bring the irrational beliefs into consciousness so that we can do something about them. The client is bringing their ideas into the light and battling them in this phase, and therefore may be invested in the irrational beliefs, and may even seek to defend them. Persistence is important here so that we are rigorous, and sometimes energetic, with our disputes.

The Ordeal can also be a useful metaphor for the client voluntarily taking on that which they perceive as dangerous to their esteem (ego) or that which they consider to be intolerably uncomfortable. That is, part of The Ordeal might involve the client going out to face little Dragons. As part of homework in REBT, we employ behavioral techniques such as flooding (Levis, 2009) as a disputation strategy and as a desensitization activity, a form of exposure to that which is feared and or deemed unbearable. The key here is to encourage the client to voluntarily expose themselves to things that they ‘cannot stand’, think are ‘awful’, think ‘must not happen’, or think ‘defines them’. As the ‘Mentor’, one of my main goals is to help the client to adapt to the world around them, rather than to change their world to suit their neuroses (e.g., by always changing A). But I would not encourage the client to just face their fears per se, I would encourage them to approach these feared stimuli with rationality and a mantra that the stimuli itself does not cause the fear, rather, it is our thoughts about the stimuli and the beliefs we hold about the stimuli that generates the fear (e.g., Turner et al., 2022). Thus, far from merely forcing themselves to Approach the Dragon’s Den to undertake the Ordeal, the client consciously engages in what they have learned thus far in REBT to inform their voluntary approach, and their internal battle.

### The reward (or seizing the treasure)

In REBT, what is the reward or treasure? In line with the Stoic tradition that REBT builds upon (Robertson, 2019; Turner, 2022), I believe that the reward or treasure is knowledge and wisdom—what is learned—and what can be utilized and passed on. This knowledge and wisdom are not only in the forms of the development of rational beliefs, but also in the development of skills such as introspection, meta-cognitive internal enquiry, disputation (cognitive change), and the realization of emotional responsibility. In line with the mythical nature of the Hero’s Journey metaphor I am constructing in this paper, this reward of knowledge and wisdom is physically manifested as gold. Smaug dwells inside a cave, greedily protecting his mountains of gold. To get the gold, we must defeat the Dragon. To fully realize our rationality, we must face our irrationality. We must weaken our irrationality to reach the gold that is our rationality. The Dragon and the gold go together like irrational and rational beliefs. Rationality is the foundation of irrationality in REBT. The *preference* becomes a demand, the *bad* becomes the awful, the *inconvenient* becomes the intolerable, the *bad behavior* becomes the bad person. The Hero slays the dragon, masters rationality, and walks out of The Ordeal with the gold. Gold is a metaphor for the sun and is pure (24-carat) and incorruptible; just like the truth and logic within the rational beliefs the client has worked so hard to develop.

The Hero (the client) seizes this treasure, in other words, develops new rational beliefs after disputing their irrational beliefs. We do not dispute the irrational beliefs and leave a void in their place. We help the client to formulate and develop rational beliefs that meet the same standards of evidence, logic, and pragmatics—this is ‘E’ stage in the GABCDE(F) framework. There is a death and a rebirth here—in that the irrational beliefs are destroyed (in reality they are weakened, but not removed) and the rational beliefs are conceived. We do not ‘replace’ irrational beliefs with rational beliefs – we provide counter arguments to irrational beliefs that are based on rationality (“I want to succeed and therefore I must” is countered with “I want to succeed, but that does not mean I must”). We can do this by using Socratic dialog. For example, we may ask “is it more rational to believe that being rejected ‘is the end of the world’, or is it more rational to believe that being rejected is ‘really bad but certainly not the end of the world’?” We can scientifically compare the current irrational beliefs to potential rational alternatives that are being developed.

Once some rational alternatives have been postulated, we need to strengthen the rational beliefs to go from intellectual insight to emotional insight (Ellis, 1963). It is one thing to understand that irrational beliefs are false, illogical, and dysfunctional, and that rational beliefs are true, logical, and functional, but it is a different thing altogether to fully endorse this viewpoint and to internalize it in day-to-day life. Like Morpheus says to Neo in The Matrix, “There is a difference between knowing the path and walking the path.” So, Seizing The Treasure is about helping the client to grasp these new rational beliefs and metacognitive skills tightly, taking them forward into the future of the real world. It is also about ensuring that the client is able to accurately reflect on what has got this to this place in their journey. What mistakes have they made that may have undercut their goal attainment? How are they now addressing these mistakes? In what ways are they now moving toward a better future? We prepare the client for the Road Back out of the introspective depths of their deep beliefs, into the ordinary world where they will need to employ rationality across the countless adversities that will (certainly but unfortunately) punctuate their lives.

### The Road Back

In REBT, the Road Back is represented by the client moving from intellectual insight, toward emotional insight. Together with the practitioner, the client must learn to strengthen and deepen their rational beliefs, continue to recognize and weaken irrational beliefs, and commit to behavioral adaptations that along with their new rational philosophy. It is important to consider that just because the client has developed some rational ideas, it does not mean that these ideas will become more deeply held beliefs. These new ideas might be abandoned the week after they are conceived—new ideas are especially susceptible to being abandoned—the client has no real connection with or commitment to them. In other words, even if you and the client have done a thorough job at disputing irrational beliefs and developing strong rational alternatives, the work is really just beginning. As tough as the Ordeal was, it is not the climax of the story, and there are further challenges ahead.

The Road Back sees the client rehearsing, challenging, refining and strengthening their rational beliefs, and of course, continuing to weaken their irrational beliefs (slaying the smaller Dragons that are seeking vengeance, perhaps). Like most roads, the Road Back is not straight and perfect, and there are some obstacles in the way, like experiencing new As that impede goal attainment, realizing new irrational beliefs that need to be addressed, or re-re-emerging resistance and doubt concerning the work and its future utility. The client will slip back into using irrational beliefs from time to time. As the practitioner (the mentor), we have an important role in keeping the client on track and helping them to meaningfully reflect on their good and bad experiences in applying REBT in their lives. As the client experiences new challenges, we continue to help them develop their fortifications by encouraging deep introspection, continuing psychoeducation concerning the rational and irrational beliefs, and boosting disputation skills and a critical and scientific approach to internal dialog. As the Shadow reappears, or as irrational beliefs continue to surface and disrupt, which they often will, the client is supported in strengthening their REBT skills.

There are many resources that can be used by the client in their own time (with the practitioner’s preparatory guidance of course) to deepen their rational beliefs, and weaken their irrational beliefs (see Turner, 2022). In this stage, the practitioner also helps the client to accept that the avoidance of As is really not a good long-term solution for emotional stability and health. Here, the client must decide if it is worth taking on the momentous task of develop a rational philosophy of life (undergirded by rational beliefs). Belief change is not easy, and for the client’s sake, we as practitioners must address their ability and motivation to persist, as part of the work we do with them. The Road Back is about the client preparing to approach their everyday lives imbued with rationality, and a host of psychological skills attained through engaging with REBT, amidst new threats to their progress.

### The resurrection

In REBT, The Resurrection is enacted when the client choses to face the critical A again (or a highly related or similar A), for the first time after our work together. I would not say that this is do or die, but it could be considered to be a figurative resurrection, in the sense that the client is revitalized with their new rationality to approach A instead of capitulating. It is also a significant event for the client and for the longevity of the REBT work that has been done. If, when faced with A, the client reacts in the same ways as they did before our work, recapitulating their unhealthy negative response, then this might undermine the work we have done. If on the other hand, the client thinks, feels and behaves in ways that are more conducive to their goals and values, with healthy negative responses, then it can reinforce the work we have done. It really helps if, during ‘the road back’, the client voluntarily and forthrightly faces some As so that they can practice their rationality and healthy responses, because this can foster the transition from intellectual insight to emotional insight (Robb et al., 1999). It also helps if we have engaged the client in techniques such as role play and or Rational Emotive (RE) imagery in order to prepare for A (see Turner, 2022). But in this resurrection phase, where the client faces the critical A, the client has the power to (maybe for the first time) think, feel, and behave in an adaptive and helpful way. If they can do this, even a little bit, their self-efficacy concerning REBT increases, their sense of control expands, and they may even experience some pride (Yes! Some positive affect!). The resurrection in the traditional Hero’s Journey is often captured with a final meeting with Death or the Nemesis. In REBT and for the client, it is facing A in the real world and choosing to prevail, using all that they have learned in their work with you. This reflects ‘F’ in the GABCDE(F) framework—the client evinces functional responses to the A, due to their rational beliefs, whereas before the journey, A was met with dysfunctional responses, at C.

### The return (with wisdom)

In REBT, The Return represents the client engaging with their normal life, but with new ideas and skills and deep philosophic change that imbues them a modified perceptual framework on their world. Thus, the practitioner must be confident that the client can apply REBT independently in their lives, and if the client experiences subsequent adversity, they can forthrightly solve their own problems. But beyond just independent remedial problem solving, the client also engages with life more functionally by proactively strengthening their rational beliefs and working continuously toward the experience of healthy emotions and behaviors. Also, the client pursues their meaningful goals (G) with the fortification to deal with the vicissitudes of life. The client should leave the work comprehensively armed with REBT principles and a commitment to exercising these principles. The client will of course face suffering. The world has not materially changed as a consequence of the client’s journey. It is still often cruel and beset with suffering and injustice. But the client *is* changed—and so the world *seems* different because their experience of the world is adjusted. New goals can be formed and pursued, what was once an A might now not be so adverse, as their rules and assumptions about themselves and the world has been adjusted.

To expand, irrational and rational beliefs can influence how we perceive the world. For example, if I perceive at A that “people judge me in social situations” and believe at B that “When people judge it shows I am a useless loser” then it likely that I will experience anxiety in social settings. But further, in social situations I am more likely to be hypervigilant to people’s eyes rolling, or awkward exchanges with people, because these features of my setting are dangerous to me. We call this attention bias (Mobini and Grant, 2007; Wenzel, 2006) and it means that I may look for and be hypervigilant toward (and even invest) the things I fear most. It can also be considered a form of confirmation bias, where I seek and even potentially imagine instances of what I fear being brought to fruition. I can creatively convince myself that people are laughing *at* me, rather than *with* me. This reinforces my irrational belief, and exacerbates my emotional turmoil, reciprocally (Turner, 2022).

The easiest option here in order to feel better quickly, is to withdraw from the situation I am in, and avoid future situations of this kind. Alternatively, if I am able to lift the irrational belief (“I am a useless loser”) and instantiate a rational belief that “Just because people judge me, it does not define who I am as a human being,” this serves to influence C and my inferences and perceptions at A. Because my self-worth is now not conditional on peoples’ judgment of me, I am less hypervigilant or biased toward embarrassing social cues; judgment is now less threatening. I now have options other than withdrawal and avoidance—I can choose to engage with people socially, or I can choose not to—it is up to me, and not determined by my fear of judgment. People may still judge me, I cannot change that, but now this judgment is met with something adaptive, rather than maladaptive, and I have a greater behavioral repertoire to draw upon. I can learn to cope with the anxiety that accompanies social exchanges and eventually may even enjoy them.

So, at the end of this momentous journey, the client is changed, in the sense that their perceptual framework has shifted and the making sense of past, present, and future is adjusted. They now set and engage with challenging goals (G) and do not avoid opportunities due to fear of or in the face of A. Belief change is not just about cognitive change. When deeply held, ubiquitous, and pervasive beliefs are shifted, it adjusts our perceptual apparatus. When we ‘return’, things appear different because we are now oriented toward opportunity and growth, rather than looking behind us for threats, we are open to and indeed actively seek challenges that stretch us. We change cognitively of course, but we also change emotionally and behaviorally.

At this point, even though the client may face adversities, they do not needlessly contribute to their own suffering. In some versions of the Hero’s Journey, the Hero returns with the Elixir, which can be taken to mean wisdom. It is worth noting that perhaps “the ultimate goal of the Hero’s Journey is for the hero to bestow the world with transformative gifts” (Allison et al., 2019), so without being too dramatic, the client may find that they are able to pass on the wisdom they have developed to people around them, and in doing so, they exercise some social utility. They can share the treasure, the gold, that they seized from the depths of the Dragon’s cave. They can share their mythos, so that others can derive meaning from it for their own lives. In any case, the work between client and practitioner can cease, and the mentor whose presence has been constant, but intermittent, can be recede. Not that we are gone forever—we can return to the client, just as Yoda returns to Luke in the Last Jedi. But with hope—the client will not need us as frequently, or at all. In the words of Voytilla (1999), “In most tales, the Return with the Elixir completes the cycle of this particular Journey. Story lines have been resolved, balance has been restored to the ordinary World, and the Hero may now embark on a new life, forever influenced by the Journey travelled.”

## Conclusion

### Why could the REBM be useful?

Readers may think I have taken leave of my senses in this article. Much of my work is highly evidence-based, and I spent my career publishing research that empirically tests REBT theory and practice. So the reader may wonder why I have authored something that is absent of data. Chiefly, I am hoping this article is at least interesting to the reader in its intellectual attempt to provide a mythological metaphor for the communication of REBT, but I also believe it has utility in our practice. We can use the REBM in the work we do, to (a) illustrate the GABCDE(F) framework to clients, patients, and fellow practitioners, (b) to help clients to make sense of the work we have done together, and (c) to teach counseling and psychotherapy students about use of narratives in their practice. Because the Hero’s Journey supposedly reflects an archetypal narrative (Campbell, 1949; Jung, 1959), nesting the GABCDE(F) framework within in it helps with the comprehension and assimilation of REBT. Some people can better connect with REBT, and most other ideas, when it is presented as a journey or story, perhaps speaking to the innateness of the monomyth. The Hero’s Journey dramatizes the client’s (and practitioner’s) experience applying REBT, giving it a narrative meaning that illustrates the importance of the work, while providing a structure that can mark progress and guide the work. In REBT, we do not meander, and we try to avoid significant tangents—just like a good story. To my knowledge, this Hero’s Journey analogy has not been used in connection with the GABCDE(F) framework in the past, so I cannot present evidence to the reader that it in some way enhances the effectiveness of REBT. All I can really say is that it helps me and the people I work with to connect with, and better understand, the REBT process.

The story-driven nature of the REBM may appeal to younger psychotherapy and coaching clients, because younger minds may feel more comfortable expressing themselves through story and metaphor (Sunderland, 2017). The alignment of REBT with the Hero’s Journey may provide opportunities for practitioners to engage younger clients who are undertaking therapy more readily because of the innate appeal of stories (Roche and Sadowsky, 2003; Storr, 2020). But as highlighted by one of the reviewers of the current paper, REBM is process-driven, requiring abstract thinking that may not be accessible to younger clients. Therefore, one vehicle through which this engagement could take place is through video gaming, because stories can be told through video games, in which players have some autonomy over the story progression. There is growing evidence that serious games (i.e., video games not just for entertainment) can have positive effects on a range of mental health outcomes (Eve et al., 2024). Indeed, recent developments in REBT offer some promising tools that integrate REBT in a video game format.

For example, REThink is an REBT-based video game in which players embark on an adventure alongside the main character ‘RETMAN’, who is a superhero who practices (REBT). The player works with RETMAN to save the minds of Earth’s inhabitants from the Irrationalizer. In the game, players develop psychological resilience through learning strategies for coping with dysfunctional negative emotions such as anxiety, anger, and depression (David D. et al., 2018). Data supports the use of REThink in children for depressive mood, negative emotions, and emotion regulation (David O. A. et al., 2018; David et al., 2019, 2021). In addition, the video game Dragon Mind (Turner and Henry, 2022) does integrate REBM into its game progression mechanisms and is a core thematic thread. In Dragon Mind, the player progresses through a story by demonstrating adaptive emotion regulation through rational thinking, they can progress through the game. All elements of the REBM are represented in Dragon Mind, which is aimed at adolescents between ages 11–16, and in the late proof of concept stages of development. However, Dragon Mind remains untested in empirical literature. The use of the Hero’s Journey in REThink is unclear, but there is perhaps scope for the game to integrate the REBM, possibly through incorporating a mentor figure, similar Obi-Wan Kenobi in Star Wars, within the Hero’s Journey, for example.

In addition to applying the REBM in more child-centered resources such as serious games, the REBM could be modified to suit younger clients. For example, the use of visual storytelling methods could be explored, such as CBT-based comic books (e.g., Fernandez and Lina, 2019), perhaps with the framing of coping as a superpower (e.g., Pimentel and DeLapp, 2022). It is possible to present the Hero’s Journey in a visual way and to analogize REBT through visual story telling. For example, in Figures 2, 3 the reader can see an example of how the Hero’s Journey can be visualized with character moments (Figure 2) and how these moments are analogous to REBT stages (Figure 3). Further, CBT-based mobile games such as the Triangle Of Life mobile app (Assigana et al., 2014) use a storybook experience with minigames to help children understand the connection between thoughts, feelings and behavior. Indeed, storybook approaches to REBT have been developed (e.g., Cristea et al., 2016). The REBM is complex and thus to suit younger clients it may need to be simplified, as is REBT when applied with children. Crucially though, to properly validate these methods of applying the REBM significant iterative co-design with children would be required to ensure that adaptations stay close to the aims of the REBM and appeal to children of varying cultural backgrounds. Indeed, the REBM has been developed in a Western (United Kingdom) context, and although the monomyth is considered to be universal, some question its universality (e.g., Brinkhof, 2024; Hambly, 2021). Therefore, to align the REBM cross-culturally more work needs to be done to ascertain whether the integration of REBT with the monomyth is useful in non-Western populations seeking psychotherapy.

The alignment between the Hero’s Journey and REBT (REBM) I have presented in this paper differs from other metaphor driven approaches to psychotherapy, like those mentioned earlier, such as TBCT. TBCT offers a specific courtroom metaphor, while REBM nests REBT within the Hero’s Journey. This affords practitioners the opportunity to tell any specific story that fits the Hero’s Journey while following the processes of REBT. For example, in the current paper I reference The Matrix and Star Wars, which are very different stories in their specifics, but similar stories in terms of their narrative alignment to the Hero’s Journey. Thus, with regards to the aims of REBT, such as deep belief change, the REBM offers a creative structure through which we can garner client engagement in the often-difficult work of understanding, challenging, and weakening dysfunctional beliefs. Destinations in the journey (i.e., monomyth stages) act as milestones, helping clients to get a sense of place in the REBT process. There is a beginning, middle, and end, like most good stories, and clients are able to understand their place in the REBT process through acknowledging their place in the Hero’s Journey. Thus, the REBM has some unique features, compared to TBCT, but of course, TBCT has garnered support in the research literature, and so the REBM must follow the same path, and researchers are encouraged to examine the test the REBM on psychological outcomes. Clearly, through empirical research we will gain an understanding of the practical implications, effectiveness, and limitations of the REBM. But more foundationally, a next step for the REBM could be to take an empirical approach to analogies drawn between the REBM and the monomyth (for possible approaches to work of this nature, see Pestana and Codina, 2019, 2020). In the current paper, I have proposed and articulated the REBM, and significant work is required to support each stage of the REBM with data. Finally, while the REBM provides a metaphorical structure for the process of REBT, one must resist the temptation or inclination to oversimply REBT or to lose the REBT goals to the goals of the monomyth.

The REBM adds specificity to the Hero’s Journey metaphor by contextualizing the monomyth within a particular psychotherapeutic framework. While the aims of REBT are not explicitly aligned with Jungian notions of wholeness and individuation, the core ideas of journeying inwards, confronting the Shadow, and prevailing through significant inner challenge is shared. Indeed, the Hero’s Journey has been used within depth therapies, and there are some insightful pieced that pertain to the Jungian perspective on the Hero’s Journey (e.g., Luton, 2020). So, clearly what I present here is not new in the sense of bringing to light the psychological important of the Hero’s Journey, but is hopefully novel in its application to REBT. New ideas in REBT are hard to achieve, and as I have discovered over the years, most of ‘my’ ideas have already been conceptualized by Ellis at some point. The more I read, the more I realize that Ellis wrote about psychology and psychotherapy very widely, and as I make sense of his writings, I delude myself less and less that I might have some original ideas about REBT. Perhaps Ellis wrote about the Hero’s Journey, perhaps not. But regardless of whether or not the ideas within this article are ‘novel’, it is my interpretation of the Hero’s Journey through the REBT lens, and I hope that it proves to be useful for practitioners attempting to apply REBT with their clients. The Hero’s Journey is considered to be an ideal metaphor for the description of psychological transformation in a person’s life (Bassil-Morozow, 2018), and in this paper I propose that it is also an ideal device for engaging clients in the difficult but ultimately rewarding process of REBT.

I will end this piece with a quote from the man who inspired this piece, Joseph Campbell, from a TV interview that aired in 1988 (Kinolorber, 2018). This quote is about Star Wars, and captures the notion that while the Hero’s Journey is often presented in film and literature as a literal journey, the meaning is deeply psychological.

“The imagery is necessarily physical and thus apparently of outer space. The inherent connotation is always, however, psychological and metaphysical, which is to say, of inner space.”

## Data Availability

The original contributions presented in the study are included in the article/supplementary material, further inquiries can be directed to the corresponding author.
